# Expression of phosphorylated ribosomal protein S6 in mesothelioma patients - correlation with clinico-pathological characteristics and outcome: results from the European Thoracic Oncology Platform (ETOP) Mesoscape project

**DOI:** 10.1038/s41379-022-01145-0

**Published:** 2022-09-17

**Authors:** Jan Hendrik Rüschoff, Martina Haberecker, Zoi Tsourti, Kristiaan Nackaerts, Marc de Perrot, Luka Brcic, Ernest Nadal, Sotirios Tsimpoukis, Steven G. Gray, Luca Ampollini, Joachim G. Aerts, Emanuela Felley-Bosco, Michaela B. Kirschner, Kim Monkhorst, Birgit Weynand, Fatemeh Bavaghar-Zaeimi, Miroslav Samarzija, Roger Llatjos, Stephen P. Finn, Enrico Silini, Jan von der Thüsen, Nesa Marti, Karerina Vervita, Roswitha Kammler, Solange Peters, Rolf A. Stahel, Paul Baas, Isabelle Opitz, for the ETOP Mesoscape consortium, Rolf Stahel, Anita Hiltbrunner, Rosita Kammler, Nesa Marti, Patrick Vagenknecht, Barbara Ruepp, Urania Dafni, Zoi Tsourti, Panagiota Zygoura, Katerina Vervita, Georgia Dimopoulou, Charitini Andriakopoulou, Androniki Stavrou, Jan H. Rüschoff, Martina Haberecker, Susanne Dettwiler, Fabiola Prutek, Christiane Mittmann

**Affiliations:** 1 Department of Pathology and Molecular Pathology, University Hospital Zurich, Zurich, Switzerland; 2 ETOP Statistical Office, Frontier Science Foundation-Hellas, Athens, Greece; 3 Department of Respiratory Oncology, KU Leuven-University Hospital Leuven, Leuven, Belgium; 4 Division of Thoracic Surgery, Toronto General Hospital, Toronto, ON Canada; 5 Diagnostic and Research Institute of Pathology, Medical University of Graz, Graz, Austria; 6 Department of Medical Oncology, Catalan Institute of Oncology, IDIBELL, L’Hospitalet, Barcelona, Spain; 7 Medical School of Athens, National and Kapodistrian University, Sotiria General Hospital, Athens, Greece; 8 Department of Clinical Medicine, St. James’s Hospital and Trinity College Dublin, Dublin, Ireland; 9 Thoracic Surgery, Department of Medicine and Surgery, University Hospital of Parma, Parma, Italy; 10 Thoracic Oncology Department, Erasmus University Medical Center, Rotterdam, Netherlands; 11 Department of Thoracic Surgery, University Hospital Zurich, Zurich, Switzerland; 12 Division of Pathology, Netherlands Cancer Institute, Amsterdam, Netherlands; 13 Department of Imaging and Pathology, University Hospitals Leuven, Leuven, Belgium; 14 Division of Thoracic Surgery, Toronto General Hospital, Toronto, QC Canada; 15 Department for Lung Diseases, University Hospital Centre Zagreb, Zagreb, Croatia; 16 Department of Pathology, Hospital Universitari de Bellvitge, L’Hospitalet, Barcelona, Spain; 17 Cancer Molecular Diagnostics and Histopathology, St. James’s Hospital and Trinity College Dublin, Dublin, Ireland; 18 Department of Pathology, Erasmus University Medical Center, Rotterdam, Netherlands; 19 Translational Research Coordination, European Thoracic Oncology Platform (ETOP), Bern, Switzerland; 20 Department of Oncology, CHUV, Lausanne University Hospital, Switzerland, Switzerland; 21 European Thoracic Oncology Platform (ETOP), Bern, Switzerland; 22 Department of Thoracic Oncology, The Netherlands Cancer Institute, Amsterdam, Netherlands; 23 ETOP Coordinating Center, Bern, Switzerland; 24 ETOP Statistical Center, Frontier Science Foundation-Hellas, Athens, Greece; 25 Mesoscape Central Laboratory: University Hospital Zürich, Zurich, Switzerland; 26 University Hospital Zürich, Zurich, Switzerland; 27 The Netherlands Cancer Institute Amsterdam, Amsterdam, Netherlands; 28 University Hospital Zürich, Zurich, Switzerland; 29 The Netherlands Cancer Institute Amsterdam, Amsterdam, Netherlands; 30 KU Leuven-University Hospital Leuven, Leuven, Belgium; 31 University Hospital of Parma, Parma, Italy; 32 University Health Network, Toronto General Hospital, Toronto, Canada; 33 University Hospital Centre Zagreb, Zagreb, Croatia; 34 Hospital Universitari de Bellvitge, Barcelona, Spain; 35 Sotiria General Hospital, Athens, Greece; 36 St. James’s Hospital and Trinity College Dublin, Dublin, Ireland; 37 Erasmus MC, Rotterdam, Netherlands

## Abstract

Pleural mesothelioma (PM) is an aggressive malignancy with poor prognosis. Although histology and pathologic stage are important prognostic factors, better prognostic biomarkers are needed. The ribosomal protein S6 is a downstream target of the phosphatidylinositol 3-kinase (PI3K) pathway involved in protein synthesis and cell proliferation. In previous studies, low phosphorylated S6 (pS6) immunoreactivity was significantly correlated with longer progression-free survival (PFS) and overall survival (OS) in PM patients. We aimed to correlate pS6 expression to clinical data in a large multi-centre PM cohort as part of the European Thoracic Oncology Platform (ETOP) Mesoscape project. Tissue Micro Arrays (TMAs) of PM were constructed and expression of pS6 was evaluated by a semi-quantitatively aggregate H-score. Expression results were correlated to patient characteristics as well as OS/PFS. pS6 IHC results of 364 patients from 9 centres, diagnosed between 1999 and 2017 were available. The primary histology of included tumours was epithelioid (70.3%), followed by biphasic (24.2%) and sarcomatoid (5.5%). TMAs included both treatment-naïve and tumour tissue taken after induction chemotherapy. High pS6 expression (181 patients with H-score>1.41) was significantly associated with less complete resection. In the overall cohort, OS/PFS were not significantly different between pS6-low and pS6-high patients. In a subgroup analysis non-epithelioid (biphasic and sarcomatoid) patients with high pS6 expression showed a significantly shorter OS (*p* < 0.001, 10.7 versus 16.9 months) and PFS (*p* < 0.001, 6.2 versus 10.8 months). In subgroup analysis, in non-epithelioid PM patients high pS6 expression was associated with significantly shorter OS and PFS. These exploratory findings suggest a clinically relevant PI3K pathway activation in non-epithelioid PM which might lay the foundation for future targeted treatment strategies.

## Introduction

Pleural mesothelioma (PM) originates from the mesothelial cell lining of the pleura surface. PM is a rare^1^ and aggressive disease with a median survival of 7 months for untreated patients^2^. There are three main histologic subtypes of PM. The epithelioid subtype is the most common (50–60%) and is known to have longer survival than sarcomatoid and biphasic PMs^3^. Most PM cases can be linked to asbestos exposure with a latency of 20–40 years^4^. Although histology and pathologic stage are important prognostic factors, there is still significant variability in the survival of patients with similar characteristics^5^. Therefore, better prognostic biomarkers are needed.

The phosphatidylinositol 3-kinase (PI3K) pathway is considered a hallmark of cancer and emerged as a potential prognostic marker^6^. Activation of the PI3K pathway is stimulated by diverse oncogenes and growth factor receptors resulting in cell growth and proliferation, making it a potential prognostic biomarker and therapeutic target^7^. The PI3K pathway consists of the PI3K, a heterodimeric lipid kinase, as well as several downstream signalling proteins including AKT, mTOR, PTEN, 4E-BP1 and ribosomal protein S6. Alteration of the PI3K pathway proteins in PM, particularly AKT and PTEN, is described in cell lines^8^. The prognostic impact of PTEN expression was also investigated in large cohorts using PM tissue^9,10^. Phosphorylated ribosomal protein S6 (pS6) expression in PM was investigated in three studies before. While two claimed pS6 immunoreactivity in PM has a prognostic impact^11,12^, a more recent study could not confirm this finding^13^.

The present study aimed to elucidate the prognostic significance of pS6 expression in a large international multi-centre cohort of PM patients, the European Thoracic Oncology Platform (ETOP) Mesoscape virtual biobank.

## Materials and methods

### Mesoscape—study design

ETOP Mesoscape was designed as an innovative platform to address the challenges of studying the molecular epidemiology of PM and to expedite our knowledge of current and evolving clinical and molecular biomarkers. As the basis of this work, a centralized biobank and clinical database were created with currently 499 cases, with most of these represented on TMAs. The ETOP Mesoscape 001 pS6 project is using health-related data and biological samples from patients, which were collected retrospectively in the framework of Mesoscape. The research was conducted according to the Mesoscape master and Mesoscape 001 pS6 substudy protocols with adherence to country-specific ethics, regulatory requirements, and Reporting Recommendations for Tumour Marker Prognostic Studies. The study was approved and waivers of consent were granted by the Ethics Committees of the participating centres. ETOP Mesoscape was performed in accordance with the Declaration of Helsinki.

#### Case selection: clinical data capture

The ETOPdata central electronic database was built for annotated comprehensive clinical data collection from each participating site on patients with PM and a minimum follow-up of 2 years or until death, whichever occurred first. According to predefined criteria, eligible patients had an adequate quantity and quality of formalin-fixed paraffin-embedded tumour for analysis and available clinical, demographic, treatment, and outcome data. To enable quality assurance of tissue analysed and pathologic staging data, it was mandatory to upload an anonymized pathology report to the ETOPdata system. All submitted data were independently medically reviewed to ensure the adequacy of clinical data.

### Study population

PM samples from 10 institutions (University Hospital Zurich, The Netherlands Cancer Institute Amsterdam, University Hospital Leuven, University Hospital of Parma, University Health Network, University Hospital Centre Zagreb, ICO Hospitalet (Bellvitge), Sotiria General Hospital, St James’s Hospital, Erasmus MC) were retrospectively collected. All patients had a histologically proven diagnosis of PM. The tumour stage was defined by tumour-node-metastasis (TNM) classification (8th edition) developed by the American Joint Commission on Cancer (AJCC) and the Union for International Cancer Control (UICC)^14,15^. Histological subclassification was done according to WHO classification^16^. Follow-up of patients was performed according to local policy.

### Tissue microarray construction and Immunohistochemistry

A total of 13 tissue microarrays (TMAs) with three to eight punches per patient were prepared at each institution and send to the central lab at the Department of Pathology and Molecular Pathology, University Hospital Zurich. TMA blocks were sectioned and stained with haematoxylin and eosin for morphologic assessment. Deparaffinised 2-μm-thick TMA sections were automatically stained with BenchMark (Ventana, Tucson, AZ) using the iView diamino benzidine detection kit (Ventana). The primary antibody was a rabbit monoclonal antibody against Phospho-S6 Ribosomal Protein (pS6, Ser240/244, Cell Signaling Technology) at a 1:50 dilution.

TMA spots with a lack of tumour tissue or damaged tissue were excluded from the analysis. Immunohistochemical evaluation of the TMAs was conducted by two independent observers (JHR, MH) in a blinded manner. The cytoplasmatic staining intensity was semi quantitatively scored 0 (negative), 1 (weak), 2 (moderate), or 3 (strong). Furthermore, the percentage of cells having any positivity was proportionally scored 0 (0%), 0.1 (1–9%), 0.5 (10–49%), or 1.0 (50% and more) as previously described^17^. The H-score was obtained by multiplying intensity with percentage staining (final range, 0 to 3, per core). The final semi-quantitative H-score was determined by averaging the H-scores of all the cores from the same patient. Slides were digitalized (Nanozoomer NDP digital slide scanner C9600-12) and scored with the Hamamatsu NDP.view 2.8.24 Software. Intratumoral heterogeneity was assessed by the deviations of the intensity scores between the cores stained for each sample. Additionally, 5% of cases were selected and compared to corresponding whole-sections.

### Statistical analysis

The analysis focused on comparing cohort characteristics and outcomes, between the cohorts of “pS6 high” versus “pS6 low” expression. The classification of patients as high/low was based on the median of the overall “H-scores” from all patients.

Differences in baseline characteristics by pS6 status were explored via the Fisher’s exact test for categorical characteristics and the Mann–Whitney test for continuous variables.

Clinical outcome was evaluated as overall survival (OS) and progression-free survival (PFS), estimated respectively, as time from diagnosis date to time to death from any cause, and time to progression/relapse or death from any cause. Median follow-up time was estimated using the reverse censoring method for OS. Both time-to-event endpoints (OS and PFS) were graphically depicted via Kaplan–Meier curves for the pS6 high and pS6 low patients, while median times and rates at 1- and 2-year time points were estimated based on the product-limit Kaplan–Meier method. Log-rank tests were performed to explore the difference in OS and PFS between pS6 high and pS6 low patients (overall as well as for prespecified groups by histology and treatment strategy). To further assess the effect of pS6 on OS/PFS, Cox proportional hazards models were fitted: univariate, as well as multivariable Cox models, adjusting for several factors of clinical interest: gender (“male” vs. “female”), ethnicity (“Caucasian” vs. “Other”), age at diagnosis (with an age cut-off of <64 and ≥64 years), ECOG performance status (“0” vs. “≥1” vs. “Unknown/Missing”), exposure to asbestos (“Yes/Possible” vs. “No” vs. “Unknown/Missing”), smoking history (“Former/Current” vs. “Never”), histology (“Epithelioid” vs. “Non-epithelioid”), localization (“Right” vs. “Left”), stage (“I” vs. “II” vs. “III” vs. “IV”) and treatment strategy (“Palliative” vs. “Complete resection”). In addition, multivariable Cox models, including also the interaction of pS6 status with each factor, were applied. The backward elimination method, with a removal criterion at 10%, was used to conclude on the significant prognostic factors. The hazard ratios (HRs) and the corresponding 95% confidence intervals (CIs) for all significant predictors are presented. The proportional hazards assumption was verified, visualizing the Schoenfeld residuals and testing the time-dependent covariates of the interaction of patients’ groups with survival time. Further exploratory subgroup analysis was performed according to the type of TMA tissue (treatment-naive or pre-treated) that was used for the assessment of pS6 expression as well as by diagnosis timing (patients diagnosed between 1999–2010 versus those diagnosed between 2011–2017). A sensitivity survival analysis was performed by splitting our cohort in three pS6 subgroups, according to 33% and 67% H-score percentiles.

Data were analysed using the SAS software package version 9.4 (SAS Institute, Cary, NC). All *p*-values (*p*) presented are 2-sided, and a *p* < 0.05 is considered statistically significant. In the case of the multiple comparisons of baseline characteristics a False Discovery Rate (FDR) adjustment was also taken into account.

## Results

### Analysis cohort

As of 9th of November 2020, 499 patients diagnosed with mesothelioma from 1999 to 2017 in 10 centres have been included in the ETOP Mesoscape database, while staining results for pS6 evaluation were available for 364 patients from 9 centres (Supplementary Tables S1–S2). Staining results were missing due to delayed tissue shipping from one centre, lack and damage of tumour tissue on the TMAs (Supplementary Fig. S1).

The pS6 analysis cohort, consisted primarily of males (86.5%) with median age at diagnosis of 64 years, while the vast majority was of Caucasian ethnicity (99.1%). Former and current smokers represented 48.0% and 11.5% of the cohort, respectively, while 40.4% were never smokers. Most of the patients (76.8%) were definitely or possibly exposed to asbestos, and for 45 of them (29.0%), asbestos fibres were detected in the lung. 114 (47.1% among those with available information) and 128 (52.9%) patients had ECOG performance status 0 and ≥1, respectively.

The majority of the cases (70.3%) were of epithelioid histology, while 24.2% were biphasic and 5.5% sarcomatoid (i.e., 29.7% non-epithelioid). The epithelioid and sarcomatoid components in biphasic PMs were evaluated in a combined score. For dubious sarcomatoid and desmoplastic PMs a cytokeratin and calretinin stain were available for cases from Zurich. Unclear external cases with a sarcomatoid/ desmoplastic histology were excluded.

In 204 (56.0%) patients tumour was detected on the right side. The distribution by clinical stage was 13.8%, 31.5%, 40.8% and 13.8% for stages I, II, III and IV respectively (based on patients with available information).

In addition, among the total 364 patients of the analysis cohort, 113 (31.0%) patients received palliative treatment as a first intention strategy (90 of them (80%) palliative chemotherapy), 249 (68.4%) patients had a macroscopic complete resection and only 2 (0.5%) patients did not receive any treatment (treatment details in Supplementary Table S3). Tumour tissue from biopsies at diagnosis and surgical resections were available. Of the 364 patients with tissue included in the pS6 analysis, 188 (51.6%) were treatment-naïve, 93 (25.5%) were pre-treated, while 83 (22.9%) could not be classified.

Baseline patient, tumour and other clinico-pathological characteristics for the pS6 analysis cohort (*n* = 364), as well as all registered Mesoscape patients (*n* = 499), are provided in Supplementary Table S4.

According to available IHC evaluations, calretinin (97.6%), CK5/6 (90.0%) and WT1 (88.6%) were detected in the vast majority of patients tested (Supplementary Table S5).

### pS6 ribosomal protein expression

**Fig. 1:**
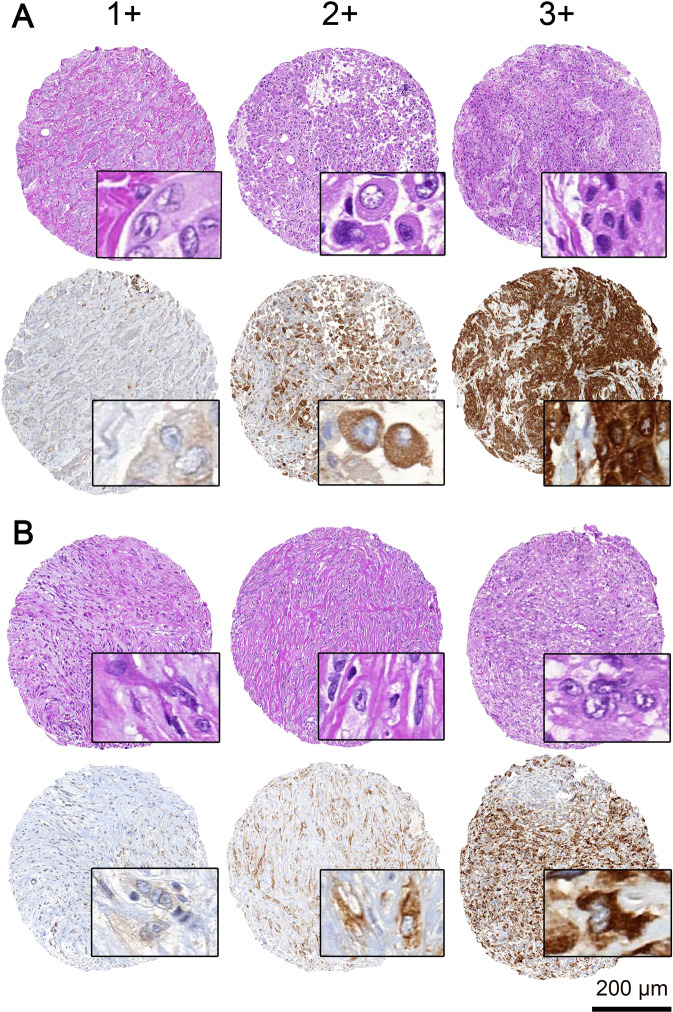
Immunohistochemical staining of pS6 ribosomal protein in pleural mesothelioma (PM). **A** Hematoxylin and eosin stained TMA punches of epithelioid PM and corresponding immunohistochemical pS6 ribosomal protein staining. The immunohistochemical staining intensity reaching from 1 (weak), 2 (moderate) to 3 (strong). **B** Hematoxylin and eosin stained TMA punches of sarcomatoid PM and corresponding immunohistochemical pS6 ribosomal protein staining. TMA tissue micro array, PM pleural mesothelioma. Scale bar 200 µm.

**Fig. 2:**
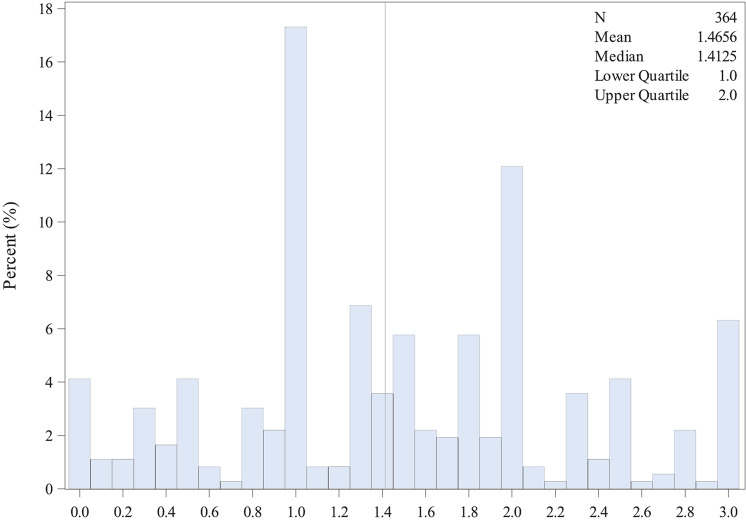
Distribution of H-scores. Grey vertical line represents the median value of H-scores.

Cytoplasmic immunohistochemical pS6 ribosomal protein expression could be evaluated in TMA cores of 364 patients (Fig. 1). The distribution of the semi-quantitative H-scores (ranging from 0 to 3) is presented in Fig. 2. The median value of H-scores, used for the classification of patients in pS6 low versus high was 1.41. Corresponding distribution by histologic subtype is available in the supplement (Fig. S2)

Furthermore, the percentage of pS6 high in the cohort of 188 treatment-naive patients was 66.0% (95% CI: 58.7–72.7%), while for the 93 pre-treated patients, pS6 high was significantly lower: 12.9% (95% CI: 6.9–21.5%) (*p* < 0.001).

### Assessment of intratumoral heterogeneity

In our cohort, 153 cases (42%) showed a homogenous intensity score among the TMA punches, 151 cases (41%) revealed a minor heterogeneity in intensity (one intensity score difference) and 60 cases (16%) had a major heterogeneity (two or three intensity scores difference) in pS6 staining. Comparison of heterogeneity evaluated in TMAs and corresponding whole sections (*n* = 19) revealed a very good level of consistency with a non-significant difference in the derived H-score (Table S6).

### Association of pS6 expression with histopathologic parameters

Patient, tumour and treatment characteristics, for the sub-cohorts of “pS6 high” versus “pS6 low” patients are presented in Table 1.

**Table 1:** Baseline characteristics, by pS6 status and overall.

Characteristic	pS6 high (*n* = 181)	pS6 low (*n* = 183)	All patients (*N* = 364)	*p*-value
Patient characteristics				
Gender - *n* (%)				
Male	156 (86.2)	159 (86.9)	315 (86.5)	0.88*
Female	25 (13.8)	24 (13.1)	49 (13.5)	
Ethnicity - *n* (%)				
African	1 (0.6)	–	1 (0.3)	–
Caucasian	168 (98.8)	174 (99.4)	342 (99.1)	
East Asian	1 (0.6)	–	1 (0.3)	
Other	–	1 (0.6)	1 (0.3)	
Unknown/Missing	11	8	19	
ECOG Performance status - *n* (%)				
0	62 (45.9)	52 (48.6)	114 (47.1)	0.86*^,~^
1	59 (43.7)	46 (43.0)	105 (43.4)	
≥2	14 (10.4)	9 (8.4)	23 (9.5)	
Unknown/Missing	46	76	122	
Smoking history - *n* (%)				
Current	18 (10.1)	23 (12.9)	41 (11.5)	0.70*^,~^
Former	88 (49.4)	83 (46.6)	171 (48.0)	
Never	72 (40.4)	72 (40.4)	144 (40.4)	
Unknown/Missing	3	5	8	
Exposure to asbestos - *n* (%)				
Yes	83 (52.5)	90 (50.6)	173 (51.5)	0.95*^,~^
Possible	39 (24.7)	46 (25.8)	85 (25.3)	0.90*^,~,$^
No	36 (22.8)	42 (23.6)	78 (23.2)	
Unknown/Missing	23	5	28	
Asbestos fibers detected in lung^*¥*^ - *n* (%)				
Yes	9 (12.3)	36 (43.9)	45 (29.0)	*–*
No	2 (2.7)	1 (1.2)	3 (1.9)	
Not tested	62 (84.9)	45 (54.9)	107 (69.0)	
Unknown/Missing	49	54	103	
Age at diagnosis (years)				
* n*	181 (100.0)	183 (100.0)	364 (100.0)	0.21^§^
Mean (95% CI)	63.7 (62.4 – 65.0)	62.7 (61.5 – 64.0)	63.2 (62.3 - 64.1)	
Median (Min-Max)	65 (35 - 89)	63 (33 - 89)	64 (33 - 89)	
Tumour characteristics				
Histology - *n* (%)				
Epithelioid	136 (75.1)	120 (65.6)	256 (70.3)	0.051*^,†^
Non-epithelioid, *incl:*	45(24.9)	63 (34.4)	108 (29.7)	0.0024^*,^^
*Biphasic*	31 (17.1)	57 (31.1)	88 (24.2)	
*Sarcomatoid*	14 (7.7)	6 (3.3)	20 (5.5)	
Localization - *n* (%)				
Right	111 (61.3)	93 (50.8)	204 (56.0)	0.034^*,&^ FDR adj: 0.14
Left	67 (37.0)	89 (48.6)	156 (42.9)	
Both	3 (1.7)	1 (0.5)	4 (1.1)	
Clinical T stage - *n* (%)				
1	23 (15.9)	31 (20.5)	54 (18.2)	0.14*^,~^
2	57 (39.3)	69 (45.7)	126 (42.6)	
3	44 (30.3)	40 (26.5)	84 (28.4)	
4	21 (14.5)	11 (7.3)	32 (10.8)	
Unknown/Missing	36	32	68	
Clinical N stage - *n* (%)				
0	101 (70.6)	97 (64.2)	198 (67.3)	0.11*^,~^
1	12 (8.4)	27 (17.9)	39 (13.3)	
2	22 (15.4)	21 (13.9)	43 (14.6)	
3	8 (5.6)	6 (4.0)	14 (4.8)	
Unknown/Missing	38	32	70	
Clinical M stage - *n* (%)				
0	138 (95.2)	145 (99.3)	283 (97.3)	0.036^*,~^ FDR adj: 0.14
1	7 (4.8)	1 (0.7)	8 (2.7)	
Unknown/Missing	36	37	73	
Clinical staging - *n* (%)				
I	15 (10.5)	25 (17.1)	40 (13.8)	0.081*^,~^
II	47 (32.9)	44 (30.1)	91 (31.5)	
III	55 (38.5)	63 (43.2)	118 (40.8)	
IV	26 (18.2)	14 (9.6)	40 (13.8)	
Unknown/Missing	38	37	75	
Treatment strategy				
Palliative	69 (38.1)	44 (24.0)	113 (31.0)	0.0032*^,#^
Complete Resection	110 (60.8)	139 (76.0)	249 (68.4)	**FDR adj: 0.039**
None	2 (1.1)	–	2 (0.5)	

(*)Fisher’s exact test, (~) Category “Unknown/Missing” is excluded, ($) Categories “Yes” and “Possible” are combined, (¥) All percentages are over the total number of patients who were exposed to asbestos (yes/possible), (§) Mann–Whitney *U* test, (†) epithelioid vs. non-epithelioid, (^) epithelioid vs. biphasic vs. sarcomatoid, (&) Category “Both” is excluded, (#) Category ‘None’ is excluded.

Statistically significant *p*-values are in bold.

The association of pS6 status with treatment strategy was found to be statistically significant. Complete resection was less common in pS6 high patients (60.8%) compared to pS6 low (76.8%) (*p* = 0.0032) (the % of pS6 high expression was 44% for patients with complete resection versus 61% for patients receiving palliative treatment).

Right side localization of the disease was also more common among pS6 high patients (61.3%) compared to 50.8% in pS6 low (the % of pS6 high expression was 43% for patients with left localization versus 54% for patients with right localization) *p* = 0.034, but we note that this observed difference was not significant after FDR adjustment. In addition, even though only 8 cases in our analysis cohort were of clinical M stage 1, an association emerges, with 7 of the 8 cases belonging to the pS6 high group (*p* = 0.036, not significant after FDR adjustment).

**Fig. 3:**
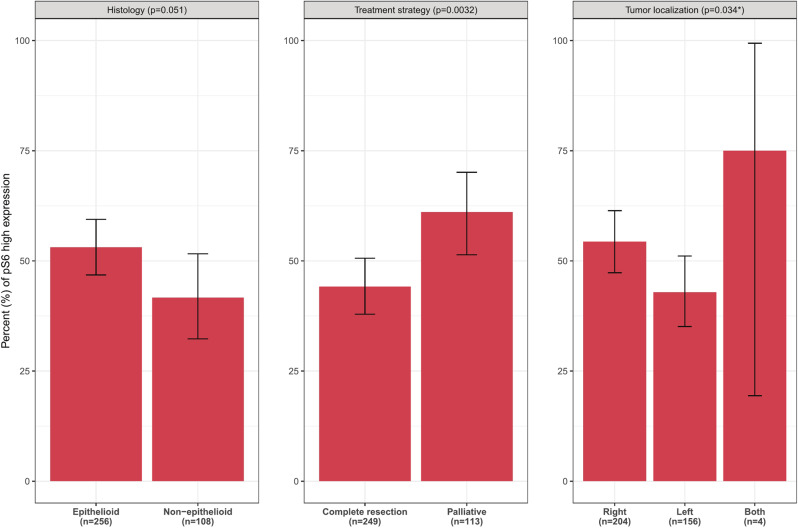
Bar plot of pS6 high prevalence by subgroups of interest. *Category “Both” is excluded. All *p*-values refer to Fisher’s exact test.

Finally, with respect to histology the observed difference was not significant (*p* = 0.051) (Fig. 3).

More particularly, among pS6 high patients, 75.1% were epithelioid and 24.9% non-epithelioid (17.1% biphasic; 7.7% sarcomatoid), versus 65.6% epithelioid and 34.4% non-epithelioid (31.1% biphasic; 3.3% sarcomatoid) for pS6 low. The percentage of pS6 high expression was 53.1% for the epithelioid and 41.7% for the non-epithelioid cases (35% in the biphasic and 70% for the few sarcomatoid cases). Overall, pS6 high expression was not significantly different between epithelioid and non-epithelioid patients (*p* = 0.051), while a significant differentiation emerges when assessing separately biphasic and sarcomatoid cases (*p* = 0.0024). Of note, in our cohort, the non-epitheloiod group primarily consists of biphasic patients.

In a post-hoc exploratοry analysis, it is found that in the more recent cases a higher percentage of pS6 high patients was detected (60% for diagnosis in 2011–2017 vs 40% in 1999–2010, *p* < 0.001).

### Prognostic value of pS6

The clinical outcome of the patients (OS, PFS) was evaluated at a median follow-up of 53.8 months (interquartile range: 43.2–77.1 months), comparable between the two sub-cohorts (*p* = 0.32). Most of the patients had died with disease (71.4%), 15.1% died without evidence of disease or with unknown disease status while only 4.7% of the patients were alive and disease-free at their last follow-up and 8.8% were alive with disease or unknown status.

A total of 155 (85.6%) deaths were observed in pS6 high patients, with median OS 18.3 months (95% CI: 16.4–20.9) and 160 (87.4%) deaths in pS6 low patients, with corresponding median OS 21.7 months (95% CI: 16.7–23.7), not significantly different (*p* = 0.52, Fig. S3). The 1-year OS estimates, along with the corresponding 95% CIs for pS6 high and low patients were 71.1% (63.9–77.2%) and 68.3% (61.0–74.5%), respectively. The OS estimates at 2-years were 37.1% (30.0–44.1%) for pS6 high patients and 41.5% (34.3–48.5%) for the pS6 low patients. Overall, the effect of pS6 was not found significant in Cox models, univariable (*p* = 0.52) or adjusting for baseline characteristics of interest (*p* = 0.28, with significant covariates: gender, histology and treatment strategy).

**Fig. 4:**
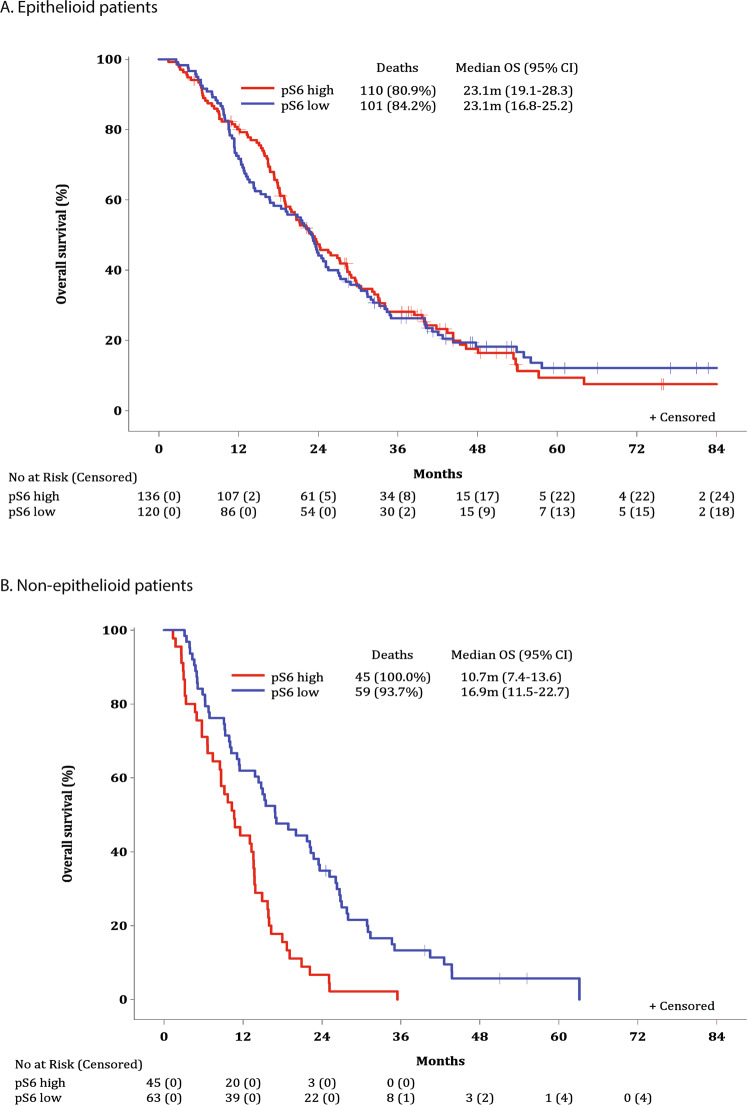
Overall survival (OS) according to histological subtype. **A** OS by pS6 status; epithelioid patients. **B** OS by pS6 status; non-epithelioid patients. Interaction *p*-value (from Cox model including pS6 status with histology interaction): <0.001. Overall median OS was 23.1 months (95% CI: 19.9–25.2) for epithelioid patients and 13.7 months (95% CI: 10.7–15.7) for non-epithelioid. Log-rank *p*-value comparing pS6 high vs. low: 0.88 for epithelioid; <0.001 for non-epithelioid.

Subgroup analysis for OS has been performed by histology (epithelioid/non-epithelioid Fig. 4) and by treatment strategy (Fig. S4A, B). A statistically significant difference in OS for the pS6 status was observed only in non-epithelioid patients (*p* < 0.001), with median OS 10.7 months (95% CI: 7.4–13.6) for pS6 high versus 16.9 months (95% CI: 11.5–22.7) for patients with pS6 low expression.

The significant effect of pS6 status in non-epithelioid patients was also verified in the multivariable Cox analysis, where the interaction of pS6 status with histology was significant (*p* < 0.001), with HR_high vs. low_ = 2.12 (95% CI: 1.42–3.17); *p* < 0.001 for the non-epithelioid group (Table 2). In addition, in both high and low pS6 groups, histology was a significant prognostic factor with favourable outcome for epithelioid patients: HR_epithelioid vs. non-epithelioid_ = 0.26 (95% CI: 0.18–0.38); *p* < 0.001 in pS6 high group and HR_epithelioid vs. non-epithelioid_ = 0.64 (95% CI: 0.46–0.89); *p* = 0.0075 in pS6 low group. Furthermore, female patients (HR_female vs. male_ = 0.55 (95% CI: 0.39–0.79); *p* = 0.0011), as well as patients who underwent complete resection (HR_complete resections vs. palliative_ = 0.50 (95% CI: 0.39–0.64); *p* < 0.001), exhibited significantly lower risk of death.

**Table 2:** Multivariable Cox proportional hazards model for overall survival (OS).

No. of patients = 362* No. of deaths = 313	Hazard Ratio	95% CI	*p*-value
Interaction effects			
pS6 status*Histology			<0.001
High vs. Low			
In Epithelioid	0.87	(0.66–1.14)	0.31
In Non-epithelioid	2.12	(1.42–3.17)	<0.001
Epithelioid vs. Non-epithelioid			
In High pS6	0.26	(0.18–0.38)	<0.001
In Low pS6	0.64	(0.46–0.89)	0.0075
Main effects			
Gender			
Female vs. Male	0.55	(0.39–0.79)	0.0011
Treatment strategy			
Complete Resection vs. Palliative	0.50	(0.39–0.64)	<0.001

(*) 2 patients who haven’t received treatment are excluded.

Note1: Variables of interest initially included in the model: Gender, Ethnicity, ECOG performance status, Smoking history, Exposure to asbestos, Age at diagnosis, Histology, Localization of tumour, Clinical staging, Treatment strategy.

Note2: *P*-values corresponding to the non-significant variables: Age at diagnosis: 0.87; Exposure to asbestos: 0.58; Clinical staging: 0.61; Ethnicity: 0.68; Smoking history: 0.54; ECOG performance status: 0.31; Localization of tumour: 0.21.

Note3: Ethnicity: Categories African, East Asian and Other are combined; Smoking history: Categories Current and Former are combined; Exposure to asbestos: Categories Yes and Possible are combined.

Analogous were the results for PFS. A total of 340 PFS events were recorded (166 (91.7%) in pS6 high patients and 174 (95.1%) in pS6 low patients, with a median PFS estimate of 12.4 months for both pS6 high and low patients and no statistically significant difference (*p* = 0.97) (Fig. S5). The PFS estimates at 1 and 2-years along with the corresponding 95% CIs for the pS6 high patients were 51.6% (44.1–58.7%) and 23.4% (17.5–29.9%), respectively. The corresponding estimates for the pS6 low patients were 50.3% (42.8–57.3%) and 21.8% (16.2–28.1%). Overall, the effect of pS6 on PFS was not found significant in Cox models, univariable (*p* = 0.97) or adjusting for baseline characteristics of interest (*p* = 0.96).

**Fig. 5:**
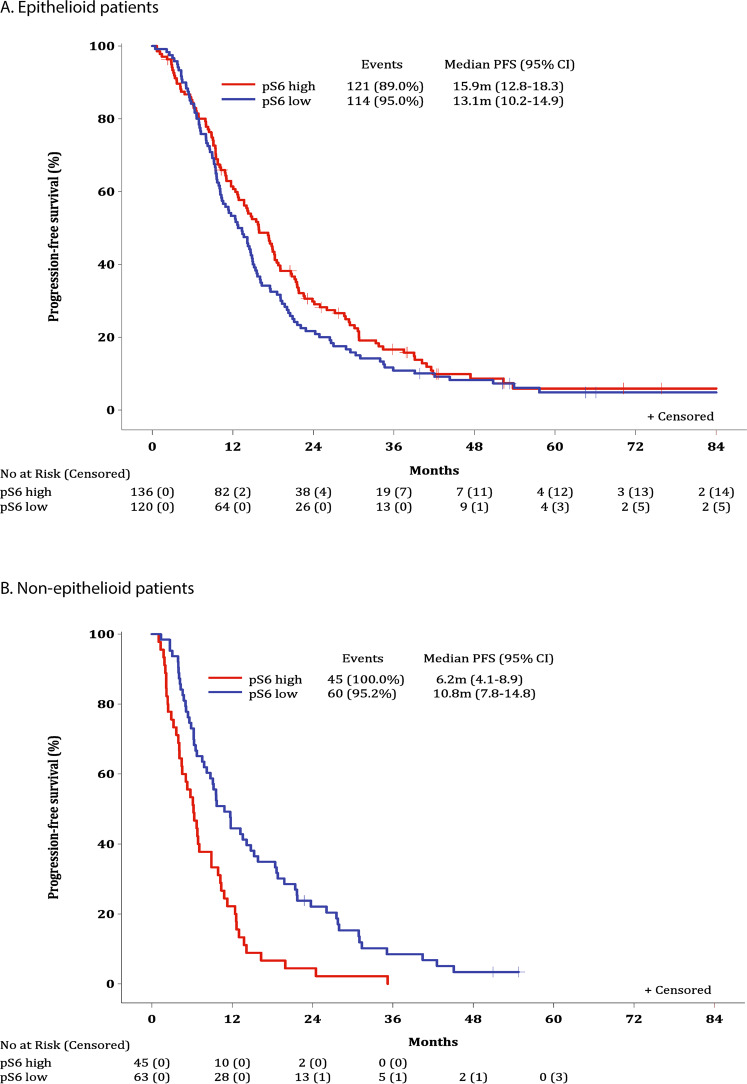
Progression-free survival (PFS) by pS6 status and histological subtype. **A** PFS by pS6 status; epithelioid patients. **B** PFS by pS6 status; non-epithelioid patients. Interaction *p*-value (from Cox model including pS6 status with histology interaction): <0.001. Overall median PFS was 14.3 months (95% CI: 12.4–15.9) for epithelioid patients and 8.8 months (95% CI: 6.3–10.4) for non-epithelioid. Log-rank *p*-value comparing pS6 high vs. low: 0.18 for epithelioid; <0.001 for non-epithelioid.

Subgroup analysis for PFS has also been performed by histology (Fig. 5A, B) and by treatment strategy (Fig. S6A, B). As with OS, a statistically significant PFS difference between high/low pS6 was observed in non-epithelioid patients (*p* < 0.001), with median PFS 6.2 months (95% CI: 4.1–8.9) for pS6 high versus 10.8 months (95% CI: 7.8–14.8) for patients with pS6 low expression.

In the multivariable Cox analysis, the interaction of pS6 status with histology was also found to be significant (*p* < 0.001), with HR_high vs. low_ = 2.06 (95% CI: 1.39–3.07); *p* < 0.001 for the non-epithelioid group, while the reverse effect on PFS was observed for the epithelioid patients; HR_high vs. low_ = 0.74 (95%CI: 0.57–0.97); *p* = 0.028 (Table 3). The favourable effect of epithelioid compared to non-epithelioid patients was detected in the group of pS6 high patients: HR_epithelioid vs. non-epithelioid_ = 0.32 (95% CI: 0.23–0.46); *p* < 0.001. In addition, significantly lower risk of PFS event was observed for female patients (HR_female vs. male_ = 0.61 [95% CI: 0.43–0.85]; *p* = 0.0034), and those who underwent complete resection (HR_complete resections vs. palliative_ = 0.55 [95% CI: 0.44–0.70]; *p* < 0.001).

**Table 3:** Multivariable Cox proportional hazards model for progression-free survival (PFS).

No. of patients = 362* No. of PFS events = 338	Hazard Ratio	95% CI	*p*-value
Interaction effects			
pS6 status*Histology			<0.001
High vs. Low			
In Epithelioid	0.74	(0.57–0.97)	0.028
In Non-epithelioid	2.06	(1.39–3.07)	<0.001
Epithelioid vs. Non-epithelioid			
In High ps6	0.32	(0.23–0.46)	<0.001
In Low ps6	0.89	(0.65–1.22)	0.48
Main effects			
Gender			
Female vs. Male	0.61	(0.43–0.85)	0.0034
Treatment strategy			
Complete Resection vs. Palliative	0.55	(0.44–0.70)	<0.001

(*) 2 patients who haven’t received treatment are excluded.

Note1: Variables of interest initially included in the model: Gender, Ethnicity, ECOG performance status, Smoking history, Exposure to asbestos, Age at diagnosis, Histology, Localization of tumour, Clinical staging, Treatment strategy.

Note2: *P*-values corresponding to the non-significant variables: Ethnicity: 0.85; Age at diagnosis: 0.50; ECOG performance status: 0.49; Exposure to asbestos: 0.53; Clinical staging: 0.33; Smoking history: 0.36; Localization of tumour: 0.22.

Note3: Ethnicity: Categories African, East Asian and Other are combined; Smoking history: Categories Current and Former are combined; Exposure to asbestos: Categories Yes and Possible are combined.

In a further exploratory subgroup analysis according to the type of TMA tissue (treatment-naive or pre-treated), the significant effect of pS6 in non-epithelioiod patients was confirmed in the subgroup of treatment-naïve tissue (*n* = 50 patients; with 26 being pS6 high), with median OS 8.6 months (95% CI: 5.7–10.8) for pS6 high versus 15.1 months (95% CI: 5.1–27.0) for pS6 low (*p* = 0.014), while in the smaller group of “pre-treated” non-epitheliod patients (37 patients; with only 5 being pS6 high) pS6 effect was not significant (*p* = 0.17). Analogous were the results for the PFS endpoint (for the 50 non-epithelioid patients with treatment-naïve tissue), with median PFS 4.5 months (95% CI: 2.4–6.1) for pS6 high versus 9.2 months (95% CI: 5.0–26.1) for pS6 low (*p* = 0.017).

In the post-hoc exploratory analysis by diagnosis timing, no significant difference was detected between the two time-groups of patients, neither for OS (medians in months (95% CI): 17.4 (14.5–21.7) vs 21.3 (18.0–24.3), log-rank *p* = 0.23) nor for PFS (medians in months: 11.8 (9.7–13.7) vs 12.8 (10.8–14.9), *p* = 0.72). In addition, the interaction effect of diagnosis time with pS6 expression was not found significant, in unadjusted (*p* = 0.78, *p* = 0.82, respectively for OS/PFS) as well as in multivariable Cox models (*p* = 0.93, 0.87).

Finally, the sensitivity survival analysis, based on three pS6 subgroups (140 cases with low pS6 H-score ≤ 1, 102 cases with intermediate pS6 H-score between 1 and 1.84, and 122 cases with high pS6 H-score ≥ 1.84) confirmed our primary findings, that is, in the overall cohort no difference in OS/PFS was found between the three pS6 subgroups (Figs. S7, S8), while the interaction with histology was significant in multivariable analysis (Tables S7, S8).

## Discussion

In the present study, the expression of pS6 was analysed in a large TMA-based PM cohort consisting of 364 patients. An overexpression of pS6 in non-epithelioid PM patients revealed a significantly shorter PFS and OS. Additionally, less complete resections were found in the pS6 high subgroup.

New prognostic biomarkers for PM are urgently needed. However, suggested prognostic biomarkers are mostly screened only on small cohorts^18–21^. In 2012 Cedres et al. reported a low pS6 immunoreactivity significantly correlated with longer PFS and OS in a relatively small cohort of 26 analysed treatment-naïve PM patients^11^. Interestingly, in 2016 Cedres et al. investigated another cohort of 23 mesotheliomas and found pAKT, FOXO3a and PD-L1 significantly associated with OS but not pS6 expression^13^. Another study by Bitanihirwe et al. detected a high pS6 expression associated with shorter PFS in a cohort of 74 treatment naïve patients^12^.

Altogether, in this study TMA-based tissue of 364 patients was included, with available clinical and outcome data, comprising epithelioid (*n* = 256, 70.3%), biphasic (*n* = 88, 24.2%) and sarcomatoid (*n* = 20, 5.5%) PMs.

All studies investigating pS6 expression in PM used the same commercially available pS6 antibody. Whereas Cedres et al.^11^ investigated the original material received for making the diagnosis, Bitanihirwe et al.^12^ and the present study investigated TMAs with at least 3 (up to 8) spots representing the tumour of each patient. For evaluation, an H-score was calculated by multiplying intensity (0–3+ ) with the corresponding percentage of positive cells. Although Cedres et al. used a continuous scale of positive cells (0–100%), in Bitanihirwe’s and this study, the percentage of positive cells was proportionally scored (0%, 1–9%, 10–49% and 50% and more). Concerning pre-treatment, Cedres and Bitanihirwe et al. only used treatment-naïve PM tissue^11,12^. Our study included TMAs consiting of treatment-naïve (*n* = 188, 51.6%) and pre-treated tumour tissue (*n* = 93, 25.5%). Bitanihirwe et al. also investigated immunhistochemical expression changes in matched pre- and postchemotherapy samples of different PI3K pathway members. A significant reduction of pS6 expression was detected after chemotherapy^12^. This finding is confirmed in our cohort where pS6 high cases occurred significantly more often in treatment-naïve (66%) than in pre-treated (12.9%) samples (*p* < 0.001).

The prognostic value of PI3K pathway members in PM is increasingly investigated. PTEN loss was associated with reduced overall survival in a large study by Opitz et al. whereas Agarwal et al. reported no relationship with survival^9,10^. Elevated pAKT expression is described in a large cohort of PM^22^, and also a relationship to longer OS was found^13^. Additionally, Cedres et al. described the expression level of FOXO3a and PD-L1 to be related to OS in PM^11,13^.

Overexpression of pS6 ribosomal protein has also been reported to be related to worse overall, shorter metastatic-free and disease-free survival in lung, ovarian and breast cancer as well as in renal cell carcinomas^23–26^.

The ribosomal protein S6 is a downstream signalling protein in the PI3K pathway involved in protein synthesis and cell proliferation^27^ and it has been recently shown to be correlated with proliferation marker ki67 in mesothelioma^28^. Although phosphorylation of S6 can also be regulated by enhanced RAS/RAF/ERK/mTORC1 activity some lines of evidence support the link between pS6 and PI3K/Akt/mTOR in mesothelioma. They include the recent finding of loss of PTEN expression in sarcomatoid mesothelioma and combined deletion of PTEN and Tp53 leds to non-epithelioid development^29^. Therefore, the PI3K pathway is also a potential therapeutic target. PM cell lines and mouse xenografts were successfully treated with a combination of CDK4/6 and PI3K/mTOR inhibitors^30^ and MET and PI3K/mTOR inhibitors^22,31^. However, treatment targeting the PI3K pathway in unselected PM patient cohorts did not bring up encouraging results up until now^32,33^.

In this study, only in non-epithelioid PMs an overexpression of pS6 was associated with shorter PFS and OS, indicating that in non-epithelioid PMs, the PI3K pathway is activated in a biologically relevant way. This activation of the PI3K pathway is further supported by findings in our previous studies, where we could show that similar to pS6 overexpression, higher expression of the upstream regulator pmTOR is also associated with shorter OS (in a histologically heterogeneous cohort), while at the same time the short survivors also show a downregulation of the pathway suppressor PTEN^9,12^. Cedres and colleagues further show an elevation of another PI3K pathway component, pAKT, however they do not observe the expected negative association with OS^11,13^. Taken together these findings are supportive of an activation of the entire pathway in sarcomatoid PM. It is known that tumours displaying sarcomatoid features (including biphasic) are associated with worse prognosis and higher chemoresistance^34,35^. Thus, new therapy options for this subtype are even more needed. Interestingly, in sarcomatoid mesotheliomas Marques et al. described a response to combined MEK and PI3K inhibition in vitro^31^. Interestingly, the RAS/MEK/ERK pathway is also known to phosphorylate pS6, however exclusively at Ser235/236^36^, not at Ser240/244, which is targeted by the antibody used in this study. Together, this might point to the consideration that the sarcomatoid subtype is a predictive biomarker for the response to targeted PI3K inhibition.

Our study has some limitations, including the retrospective approach. The TMAs consisted of 3 to 8 punches per patient which might not fully represent the heterogeneous nature of each tumour. However, an internal comparison of TMAs to corresponding whole slides revealed H-scores with a non-significant difference. Although a significant higher percentage of pS6 high patients was detected in more recent cases (1999–2010 vs 2011–2017, *p* < 0.001), no interaction effect of diagnosis timing and pS6 expression on the clinical outcome was detected.

To the best of our knowledge, this study represents the largest immunohistochemical screening of mesothelioma specimens for the expression of pS6 ribosomal protein with correlation to clinical data. In the primary analysis of all-histologies cohort no association of pS6 expression with outcome is detected. However, subgroup analysis indicates high pS6 expression as a prognostic biomarker associated with significantly shorter OS and PFS in non-epithelioid PM patients.

These exploratory, hypothesis-generating, results suggest a relevant PI3K pathway activation in non-epithelioid PM which might lay the foundations for future targeted treatment strategies.

## Supplementary information

Supplementary Tables and Figures

## Data Availability

Access to de-identified individual participant data used in this study may be requested by researchers by submitting a research proposal (Mesoscape@etop-eu.org), which will be reviewed for scientific merit and feasibility in accordance with the ETOP Biobank Policy.
