# Correction to: Expression of phosphorylated ribosomal protein S6 in mesothelioma patients - correlation with clinico-pathological characteristics and outcome: results from the European Thoracic Oncology Platform (ETOP) Mesoscape project

**DOI:** 10.1038/s41379-022-01170-z

**Published:** 2022-10-17

**Authors:** Jan Hendrik Rüschoff, Martina Haberecker, Zoi Tsourti, Kristiaan Nackaerts, Marc de Perrot, Luka Brcic, Ernest Nadal, Sotirios Tsimpoukis, Steven G. Gray, Luca Ampollini, Joachim G. Aerts, Emanuela Felley-Bosco, Michaela B. Kirschner, Kim Monkhorst, Birgit Weynand, Fatemeh Bavaghar-Zaeimi, Miroslav Samarzija, Roger Llatjos, Stephen P. Finn, Enrico Silini, Jan von der Thüsen, Nesa Marti, Karerina Vervita, Roswitha Kammler, Solange Peters, Rolf A. Stahel, Paul Baas, Isabelle Opitz, for the ETOP Mesoscape consortium, Rolf Stahel, Anita Hiltbrunner, Rosita Kammler, Nesa Marti, Patrick Vagenknecht, Barbara Ruepp, Urania Dafni, Zoi Tsourti, Panagiota Zygoura, Katerina Vervita, Georgia Dimopoulou, Charitini Andriakopoulou, Androniki Stavrou, Jan H. Rüschoff, Martina Haberecker, Susanne Dettwiler, Fabiola Prutek, Christiane Mittmann

**Affiliations:** 1 Department of Pathology and Molecular Pathology, University Hospital Zurich, Zurich, Switzerland; 2 ETOP Statistical Office, Frontier Science Foundation-Hellas, Athens, Greece; 3 Department of Respiratory Oncology, KU Leuven-University Hospital Leuven, Leuven, Belgium; 4 Division of Thoracic Surgery, Toronto General Hospital, Toronto, ON Canada; 5 Diagnostic and Research Institute of Pathology, Medical University of Graz, Graz, Austria; 6 Department of Medical Oncology, Catalan Institute of Oncology, IDIBELL, L’Hospitalet, Barcelona, Spain; 7 Medical School of Athens, National and Kapodistrian University, Sotiria General Hospital, Athens, Greece; 8 Department of Clinical Medicine, St. James’s Hospital and Trinity College Dublin, Dublin, Ireland; 9 Thoracic Surgery, Department of Medicine and Surgery, University Hospital of Parma, Parma, Italy; 10 Thoracic Oncology Department, Erasmus University Medical Center, Rotterdam, Netherlands; 11 Department of Thoracic Surgery, University Hospital Zurich, Zurich, Switzerland; 12 Division of Pathology, Netherlands Cancer Institute, Amsterdam, Netherlands; 13 Department of Imaging and Pathology, University Hospitals Leuven, Leuven, Belgium; 14 Division of Thoracic Surgery, Toronto General Hospital, Toronto, QC Canada; 15 Department for Lung Diseases, University Hospital Centre Zagreb, Zagreb, Croatia; 16 Department of Pathology, Hospital Universitari de Bellvitge, L’Hospitalet, Barcelona, Spain; 17 Cancer Molecular Diagnostics and Histopathology, St. James’s Hospital and Trinity College Dublin, Dublin, Ireland; 18 Department of Pathology, Erasmus University Medical Center, Rotterdam, Netherlands; 19 Translational Research Coordination, European Thoracic Oncology Platform (ETOP), Bern, Switzerland; 20 Department of Oncology, CHUV, Lausanne University Hospital, Switzerland, Switzerland; 21 European Thoracic Oncology Platform (ETOP), Bern, Switzerland; 22 Department of Thoracic Oncology, The Netherlands Cancer Institute, Amsterdam, Netherlands; 23 ETOP Coordinating Center, Bern, Switzerland; 24 ETOP Statistical Center, Frontier Science Foundation-Hellas, Athens, Greece; 25 Mesoscape Central Laboratory: University Hospital Zürich, Zurich, Switzerland; 26 University Hospital Zürich, Zurich, Switzerland; 27 The Netherlands Cancer Institute Amsterdam, Amsterdam, Netherlands; 28 University Hospital Zürich, Zurich, Switzerland; 29 The Netherlands Cancer Institute Amsterdam, Amsterdam, Netherlands; 30 KU Leuven-University Hospital Leuven, Leuven, Belgium; 31 University Hospital of Parma, Parma, Italy; 32 University Health Network, Toronto General Hospital, Toronto, Canada; 33 University Hospital Centre Zagreb, Zagreb, Croatia; 34 Hospital Universitari de Bellvitge, Barcelona, Spain; 35 Sotiria General Hospital, Athens, Greece; 36 St. James’s Hospital and Trinity College Dublin, Dublin, Ireland; 37 Erasmus MC, Rotterdam, Netherlands

**Keywords:** Respiratory tract diseases, Mesothelioma, Prognostic markers

Correction to: *Modern Pathology* 10.1038/s41379-022-01145-0, published online 17 September 2022

The original version of this article unfortunately contained an error in an author name. A pathologist within the consortium, Dr. Letizia Gnetti from Parma, Italy noticed that her name was missing one letter. In lieu of ‘Gnett’ it should be ‘Gnetti’. The original article has been corrected.

