# Supplementary material for: Expression of phosphorylated ribosomal protein S6 in mesothelioma patients - correlation with clinico-pathological characteristics and outcome: results from the European Thoracic Oncology Platform (ETOP) Mesoscape project
Source: Mod Pathol. 2022 Sep 17;35(12):1888–99. doi: 10.1038/s41379-022-01145-0 (PMC9708564; doi:10.1038/s41379-022-01145-0)
Supplement: Supplementary file 1 — Supplementary Tables and Figures [file 41379_2022_1145_MOESM1_ESM.docx]

**SUPPLEMENT FOR**

**Expression of phosphorylated ribosomal protein S6 in mesothelioma patients -correlation with clinico-pathological characteristics and outcome: results from the European Thoracic Oncology Platform (ETOP) Mesoscape**

**List of Supplementary Tables**

**Table S1:** Availability of pS6 results by center

**Table S2:** Availability of results by year of diagnosis

**Table S3:** Treatment information

**Table S4:** Baseline characteristics, for all Mesoscape patients and patients with available pS6

**Table S5:** Immunohistochemistry (IHC) staining, by pS6 status and overall

**Table S6:** Comparison of heterogeneity between TMAs and whole section evaluation

**Table S7:** Multivariable Cox proportional hazards model for overall survival (OS), based on 3-level pS6 grouping

**Table S8:** Multivariable Cox proportional hazards model for progression-free survival (PFS), based on 3-level pS6 grouping

**List of Supplementary Figures**

**Figure S1.** Flowchart of the Mesoscape pS6 study

**Figure S2.** Distribution of H-scores by histologic subtype

**Figure S3:** Overall survival (OS), by pS6 status

**Figure S4A:** Overall survival (OS), by pS6 status; palliative treatment

**Figure S4B:** Overall survival (OS), by pS6 status; complete resection

**Figure S5:** Progression-free survival (PFS), by pS6 status

**Figure S6A:** Progression-free survival (PFS), by pS6 status; palliative treatment

**Figure S6B:** Progression-free survival (PFS), by pS6 status; complete resection

**Figure S7:** Overall survival (OS), by 3-level pS6 status

**Figure S8:** Progression-free survival (PFS), by 3-level pS6 status

**TABLES**

**Table S1:** Availability of pS6 results by center

| **Center** | **N (%) of patients with pS6 results** |
| --- | --- |
| CHE005 University Hospital Zurich | 223 (61.3) |
| BEL015 University Hospital Leuven | 39 (10.7) |
| ITA200 University Hospital of Parma | 6 (1.6) |
| CAN328 University Health Network | 28 (7.7) |
| CRO199 University Hospital Centre Zagreb | 27 (7.4) |
| ESP059 ICO Hospitalet (Bellvitge) | 19 (5.2) |
| GRC039 Sotiria General Hospital | 10 (2.7) |
| IRL004 St James's Hospital | 9 (2.5) |
| NLD203 Erasmus MC | 3 (0.8) |
| **Total patients** | **364 (100.0)** |

**Table S2:** Availability of results by year of diagnosis

| **Year of diagnosis** | **N (%) of patients with pS6 results** |
| --- | --- |
| 1999 | 8 (2.2) |
| 2000 | 10 (2.7) |
| 2001 | 8 (2.2) |
| 2002 | 8 (2.2) |
| 2003 | 13 (3.6) |
| 2004 | 18 (4.9) |
| 2005 | 18 (4.9) |
| 2006 | 17 (4.7) |
| 2007 | 21 (5.8) |
| 2008 | 17 (4.7) |
| 2009 | 19 (5.2) |
| 2010 | 30 (8.2) |
| 2011 | 35 (9.6) |
| 2012 | 46 (12.6) |
| 2013 | 32 (8.8) |
| 2014 | 33 (9.1) |
| 2015 | 25 (6.9) |
| 2016 | 5 (1.4) |
| 2017 | 1 (0.3) |
| **Total patients** | **364 (100.0)** |

**Table S3:** Treatment information

| **A. Palliative treatment** | | **All patients (N=113)** | |
| --- | --- | --- | --- |
| **Palliative Chemotherapy - n (%)** | | | |
| Yes | | 90 (79.6) | |
| No | | 22 (19.5) | |
| Unknown/Missing | | 1 (0.9) | |
| **Palliative Radiotherapy - n (%)** | | | |
| Yes | | 18 (15.9) | |
| No | | 86 (76.1) | |
| Unknown/Missing | | 9 (8.0) | |
| **B. Complete resection** | **All patients (N=249)** | |  |
| **Status of resections – n (%)** |  | |  |
| MCR (Macroscopic Complete Resection) | 198 (79.5) | |  |
| R2 | 31 (12.4) | |  |
| Not applicable | 20 (8.1) | |  |

**Table S4:** Baseline characteristics, for all Mesoscape patients and patients with available pS6

| **Characteristic** | | **pS6 available (n=364)** | **pS6 not available (n=135)** | | **All Mesoscape patients (N=499)** | **p-value** |
| --- | --- | --- | --- | --- | --- | --- |
| **Patient Characteristics** | | | | | | |
| **Gender - n (%)** | | | | | | |
| Male | | 315 (86.5) | 106 (78.5) | | 421 (84.4) | 0.037*  ***FDR adj: 0.11*** |
| Female | | 49 (13.5) | 29 (21.5) | | 78 (15.6) |  |
| **Ethnicity - n (%)** | | | | | | |
| African | | 1 (0.3) | 1 (0.8) | | 2 (0.4) | - |
| Caucasian | | 342 (99.1) | 130 (98.5) | | 472 (99.0) |  |
| East Asian | | 1 (0.3) | - | | 1 (0.2) |  |
| Other | | 1 (0.3) | 1 (0.8) | | 2 (0.4) |  |
| Unknown/Missing | | 19 | 3 | | 22 |  |
| **ECOG Performance status - n (%)** | | | | | | |
| 0 | | 114 (47.1) | 52 (42.3) | | 166 (45.5) | 0.31*^,~^ |
| 1 | | 105 (43.4) | 63 (51.2) | | 168 (46.0) |  |
| ≥ 2 | | 23 (9.5) | 8 (6.5) | | 31 (8.5) |  |
| Unknown/Missing | | 122 | 12 | | 134 |  |
| **Smoking history - n (%)** | | | | | | |
| Current | | 41 (11.5) | 12 (9.2) | | 53 (10.9) | 0.32*^,~^ |
| Former | | 171 (48.0) | 56 (42.7) | | 227 (46.6) |  |
| Never | | 144 (40.4) | 63 (48.1) | | 207 (42.5) |  |
| Unknown/Missing | | 8 | 4 | | 12 |  |
| **Exposure to asbestos - n (%)** | | | | | | |
| Yes | | 173 (51.5) | 65 (50.8) | | 238 (51.3) | 0.58*^,~^ |
| Possible | | 85 (25.3) | 28 (21.9) | | 113 (24.4) | 0.40*^,~,$^ |
| No | | 78 (23.2) | 35 (27.3) | | 113 (24.4) |  |
| Unknown/Missing | | 28 | 7 | | 35 |  |
| **Asbestos fibers detected in lung^¥^ - n (%)** | | | | | | |
| Yes | | 45 (29.0) | 5 (11.6) | | 50 (25.3) | - |
| No | | 3 (1.9) | 1 (2.3) | | 4 (2.0) |  |
| Not tested | | 107 (69.0) | 37 (86.0) | | 144 (72.7) |  |
| Unknown/Missing | | 103 | 50 | | 153 |  |
| **Age at diagnosis (years)** | | | | | | |
| n | | 364 (100.0) | 135 (100.0) | | 499 (100.0) | 0.0503^§^  ***FDR adj: 0.12*** |
| Mean (95% CI) | | 63.2 (62.3 - 64.1) | 65.1 (63.7 - 66.6) | | 63.7 (63.0 - 64.5) |  |
| Median (Min-Max) | | 64 (33 - 89) | 65 (35 - 88) | | 64 (33 - 89) |  |
| **Tumour characteristic** | | | | | | |
| **Histology - n (%)** | | | | | | |
| Epithelioid | 256 (70.3) | | | 105 (77.8) | 361 (72.3) | 0.11*^,†^ |
| Non-epithelioid, incl: | 108 (29.7) | | | 30 (22.2) | 138 (27.7) | 0.11*^,^^ |
| *Biphasic* | *88 (24.2)* | | | *21 (15.6)* | *109 (21.8)* |  |
| *Sarcomatoid* | *20 (5.5)* | | | *9 (6.7)* | *29 (5.8)* |  |
| **Localization - n (%)** | | | | | | |
| Right | 204 (56.0) | | | 91 (67.4) | 295 (59.1) | 0.022* |
| Left | 156 (42.9) | | | 41 (30.4) | 197 (39.5) | 0.017*^,&^ |
| Both | 4 (1.1) | | | 3 (2.2) | 7 (1.4) | **FDR adj***^,&^ ***0.072*** |
| **Clinical T stage - n (%)** | | | | | | |
| 1 | 54 (18.2) | | | 16 (15.8) | 70 (17.6) | 0.33*^,~^ |
| 2 | 126 (42.6) | | | 35 (34.7) | 161 (40.6) |  |
| 3 | 84 (28.4) | | | 35 (34.7) | 119 (30.0) |  |
| 4 | 32 (10.8) | | | 15 (14.9) | 47 (11.8) |  |
| Unknown/Missing | 68 | | | 34 | 102 |  |
| **Clinical N stage - n (%)** | | | | | | |
| 0 | 198 (67.3) | | | 67 (67.7) | 265 (67.4) | 0.059*^,~^ |
| 1 | 39 (13.3) | | | 5 (5.1) | 44 (11.2) |  |
| 2 | 43 (14.6) | | | 22 (22.2) | 65 (16.5) |  |
| 3 | 14 (4.8) | | | 5 (5.1) | 19 (4.8) |  |
| Unknown/Missing | 70 | | | 36 | 106 |  |
| **Clinical M stage - n (%)** | | | | | | |
| 0 | 283 (97.3) | | | 90 (90.9) | 373 (95.6) | 0.018*^,~^ |
| 1 | 8 (2.7) | | | 9 (9.1) | 17 (4.4) | ***FDR adj:*** |
| Unknown/Missing | 73 | | | 36 | 109 | ***0.072*** |
| **Clinical staging - n (%)** | | | | | | |
| I | 40 (13.8) | | | 13 (13.7) | 53 (13.8) | 0.18*^,~^ |
| II | 91 (31.5) | | | 20 (21.1) | 111 (28.9) |  |
| III | 118 (40.8) | | | 43 (45.3) | 161 (41.9) |  |
| IV | 40 (13.8) | | | 19 (20.0) | 59 (15.4) |  |
| Unknown/Missing | 75 | | | 40 | 115 |  |
| **Treatment strategy** | | | | | | |
| Palliative | 113 (31.0) | | | 91 (67.4) | 204 (40.9) | <0.001*^,#^ |
| Complete Resection | 249 (68.4) | | | 43 (31.9) | 292 (58.5) | ***FDR adj:*** |
| None | 2 (0.5) | | | 1 (0.7) | 3 (0.6) | ***<0.001*** |

*(*) Fisher's exact test, (~) Category 'Unknown/Missing' is excluded, ($) Categories 'Yes' and 'Possible' are combined, (¥) All percentages are over the total number of patients who were exposed to asbestos (yes/possible), (§) Mann-Whitney U test, (†) epithelioid vs. non-epithelioid, (^) epithelioid vs. biphasic vs. sarcomatoid, (&) Category 'Both' is excluded, (#) Category ‘None’ is excluded*

**Table S5:** Immunohistochemistry (IHC) staining, by pS6 status and overall

| **IHC staining** | **pS6 high (n=181)** | **pS6 low (n=183)** | **All patients (N=364)** | **p-value** |
| --- | --- | --- | --- | --- |
| **Calretinin - n (%)** | | | | |
| Positive | 154 (98.1) | 125 (96.9) | 279 (97.6) | 0.70*^,~^ |
| Negative | 3 (1.9) | 4 (3.1) | 7 (2.4) |  |
| Not tested | 24 | 54 | 78 |  |
| **CK5/6 - n (%)** | | | | |
| Positive | 107 (87.7) | 73 (93.6) | 180 (90.0) | 0.23*^,~^ |
| Negative | 15 (12.3) | 5 (6.4) | 20 (10.0) |  |
| Not tested | 59 | 105 | 164 |  |
| **Pan-CK - n (%)** | | | | |
| Positive | 30 (88.2) | 51 (100.0) | 81 (95.3) | ***0.023*^,~^*** |
| Negative | 4 (11.8) | - | 4 (4.7) | ***FDR adj:*** |
| Not tested | 147 | 132 | 279 | ***0.14*** |
| **TTF1 - n (%)** | | | | |
| Negative | 111 (100.0) | 77 (100.0) | 188 (100.0) | - |
| Not tested | 70 | 106 | 176 |  |
| **Ber-EP4 - n (%)** | | | | |
| Positive | 9 (11.5) | 14 (15.9) | 23 (13.9) | 0.50*^,~^ |
| Negative | 69 (88.5) | 74 (84.1) | 143 (86.1) |  |
| Not tested | 103 | 95 | 198 |  |
| **WT1 - n (%)** | | | | |
| Positive | 85 (87.6) | 71 (89.9) | 156 (88.6) | 0.81*^,~^ |
| Negative | 12 (12.4) | 8 (10.1) | 20 (11.4) |  |
| Not tested | 84 | 104 | 188 |  |
| **MOC-31 - n (%)** | | | | |
| Negative | 1 (100.0) | 2 (100.0) | 3 (100.0) | - |
| Not tested | 180 | 181 | 361 |  |
| **D2-40 - n (%)** | | | | |
| Positive | 30 (90.9) | 30 (90.9) | 60 (90.9) | >0.99*^,~^ |
| Negative | 3 (9.1) | 3 (9.1) | 6 (9.1) |  |
| Not tested | 148 | 150 | 298 |  |
| **CEA - n (%)** | | | | |
| Positive | 2 (2.1) | 1 (1.2) | 3 (1.7) | - |
| Negative | 94 (97.9) | 84 (98.8) | 178 (98.3) |  |
| Not tested | 85 | 98 | 183 |  |

*(*) Fisher's exact test, (~) Category 'Not tested' is excluded*

**Table S6:** Comparison of heterogeneity between TMAs and whole section evaluation

| **Intensity scores on TMAs** | **Whole sections results** | | |
| --- | --- | --- | --- |
|  | Homogenous | Minor Heterogeneity | Major Heterogeneity |
| Homogenous (n=10) | 5 (50%) | 5 (50%) | 0 (0%) |
| Minor Heterogeneity (n=5) | 0 (0%) | 5 (100%) | 0 (0%) |
| Major Heterogeneity (n=4) | 0 (0%) | 0 (0%) | 4 (100%) |
| **Differences in H-score (Whole sections – TMAs) – n (% over 19 cases)**  0 – 9 (47%)  0.25 – 5 (26%)  0.35 – 2 (11%)  0.4 - 1 (5%)  0.75 – 2 (11%)  Mean H-score difference: 0.0605 (p=0.41, Paired Wilcoxon signed rank test) | | | |

**Table S7:** Multivariable Cox proportional hazards model for overall survival (OS), based on 3-level pS6 grouping

| **No. of patients=362***  **No. of deaths=313** | **Hazard Ratio** | **95% CI** | **p-value** |
| --- | --- | --- | --- |
| **Interaction effects** | | | |
| **pS6 status*Histology** |  |  | 0.071 |
| **In Epithelioid** |  |  |  |
| High vs. Intermediate | 1.07 | (0.77 - 1.50) | 0.68 |
| High vs. Low | 0.99 | (0.71 - 1.37) | 0.94 |
| Intermediate vs. Low | 0.92 | (0.66 - 1.29) | 0.63 |
| **In Non-Epithelioid** |  |  |  |
| High vs. Intermediate | 1.43 | (0.81 - 2.51) | 0.21 |
| High vs. Low | 1.89 | (1.19 - 2.99) | 0.0065 |
| Intermediate vs. Low | 1.32 | (0.79 - 2.20) | 0.28 |
| **Epithelioid vs. Non-epithelioid** |  |  |  |
| In High ps6 | 0.31 | (0.20 - 0.48) | <0.001 |
| In Intermediate ps6 | 0.41 | (0.25 - 0.68) | <0.001 |
| In Low ps6 | 0.59 | (0.41 - 0.85) | 0.0045 |
| **Main effects** | | | |
| **Gender** | | | |
| Female vs. Male | 0.55 | (0.39 - 0.79) | 0.0011 |
| **Treatment strategy** | | | |
| Complete Resection vs. Palliative | 0.52 | (0.41 - 0.66) | <0.001 |

*(*) 2 patients who haven’t received treatment are excluded*

***Note1:*** *Variables of interest initially included in the model: Gender, Ethnicity, ECOG performance status, Smoking history, Exposure to asbestos, Age at diagnosis, Histology, Localization of tumour, Clinical staging, Treatment strategy.*

***Note2:*** *P-values corresponding to the non-significant variables: Age at diagnosis: 0.81; Ethnicity: 0.61; Clinical staging: 0.63; Exposure to asbestos: 0.67; Smoking history: 0.52; ECOG performance status: 0.28; Localization of tumour: 0.14.*

***Note3:*** *Ethnicity: Categories African, East Asian and Other are combined; Smoking history: Categories Current and Former are combined; Exposure to asbestos: Categories Yes and Possible are combined.*

**Table S8:** Multivariable Cox proportional hazards model for progression-free survival (PFS), based on 3-level pS6 grouping

| **No. of patients=362***  **No. of PFS events=338** | **Hazard Ratio** | **95% CI** | **p-value** |
| --- | --- | --- | --- |
| **Interaction effects** | | | |
| **pS6 status*Histology** |  |  | 0.0055 |
| **In Epithelioid** |  |  |  |
| High vs. Intermediate | 1.01 | (0.73 - 1.39) | 0.96 |
| High vs. Low | 0.74 | (0.55 - 1.01) | 0.062 |
| Intermediate vs. Low | 0.74 | (0.53 - 1.02) | 0.065 |
| **In Non-Epithelioid** |  |  |  |
| High vs. Intermediate | 1.48 | (0.84 - 2.59) | 0.18 |
| High vs. Low | 1.81 | (1.15 - 2.86) | 0.011 |
| Intermediate vs. Low | 1.23 | (0.74 - 2.04) | 0.43 |
| **Epithelioid vs. Non-epithelioid** |  |  |  |
| In High ps6 | 0.36 | (0.24 - 0.55) | <0.001 |
| In Intermediate ps6 | 0.53 | (0.32 - 0.87) | 0.011 |
| In Low ps6 | 0.88 | (0.62 - 1.25) | 0.47 |
| **Main effects** | | | |
| **Gender** | | | |
| Female vs. Male | 0.61 | (0.44 - 0.85) | 0.004 |
| **Treatment strategy** | | | |
| Complete Resection vs. Palliative | 0.57 | (0.45 - 0.72) | <0.001 |

*(*) 2 patients who haven’t received treatment are excluded*

***Note1:*** *Variables of interest initially included in the model: Gender, Ethnicity, ECOG performance status, Smoking history, Exposure to asbestos, Age at diagnosis, Histology, Localization of tumour, Clinical staging, Treatment strategy.*

***Note2:*** *P-values corresponding to the non-significant variables: Ethnicity: 0.79; Clinical staging: 0.65; Age at diagnosis: 0.53; ECOG PS: 0.46; Exposure to asbestos: 0.42; Smoking history: 0.25; Localization of tumour: 0.17.*

***Note3:*** *Ethnicity: Categories African, East Asian and Other are combined; Smoking history: Categories Current and Former are combined; Exposure to asbestos: Categories Yes and Possible are combined.*


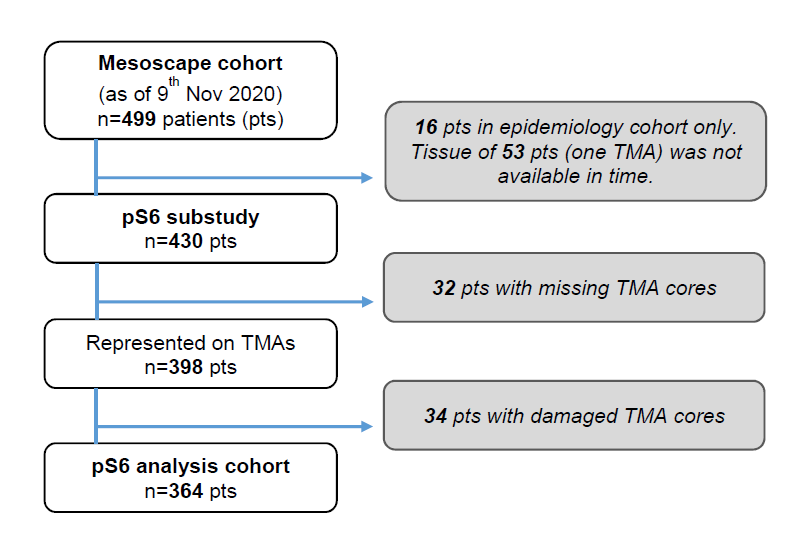
**Figure S1:** Flowchart of Mesoscape pS6 study


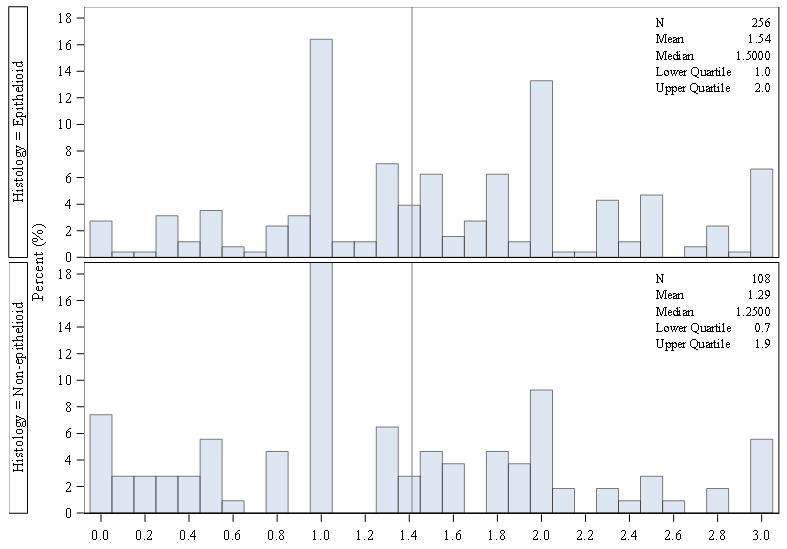


**Figure S2:** Distribution of H-scores by histologic subtype

***Note:*** *Grey vertical line represents the median value of H-scores.*


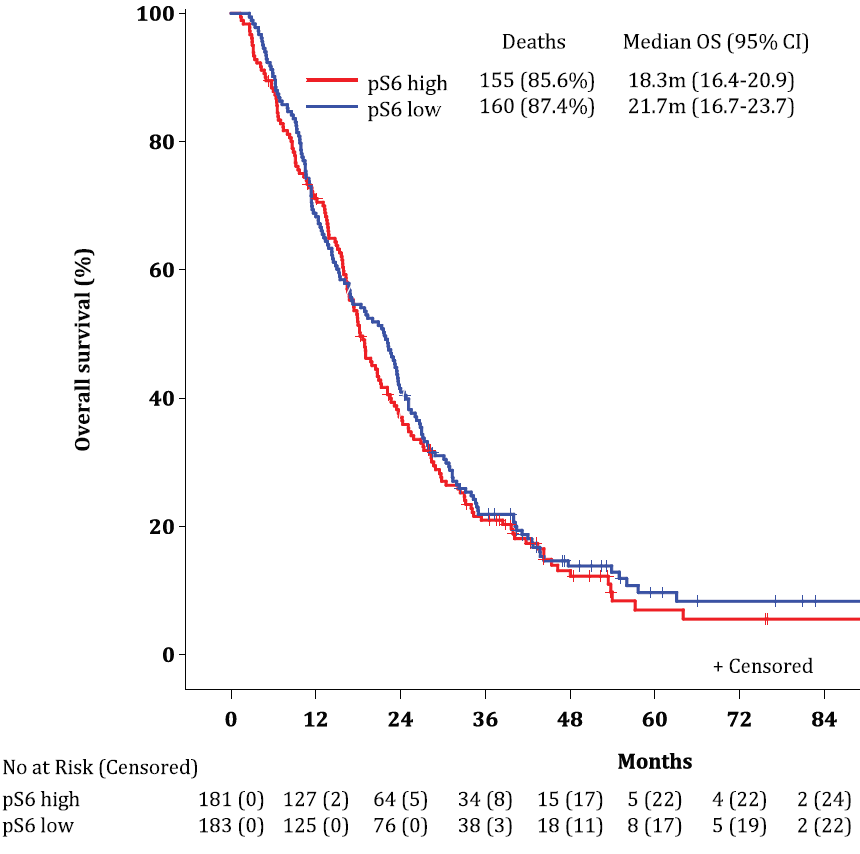


**Figure S3:** Overall survival (OS), by pS6 status

***Note:*** *Log-rank p-value=0.52*


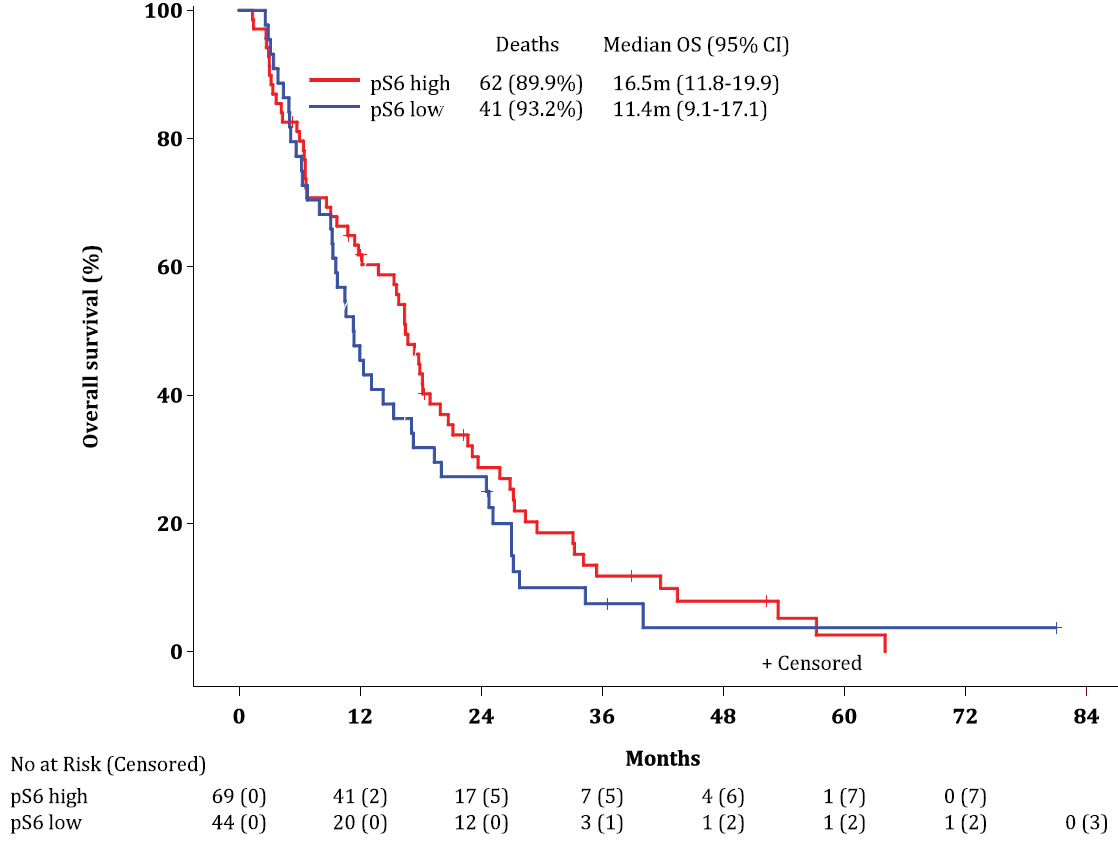


**Figure S4A:** Overall survival (OS), by pS6 status; palliative treatment


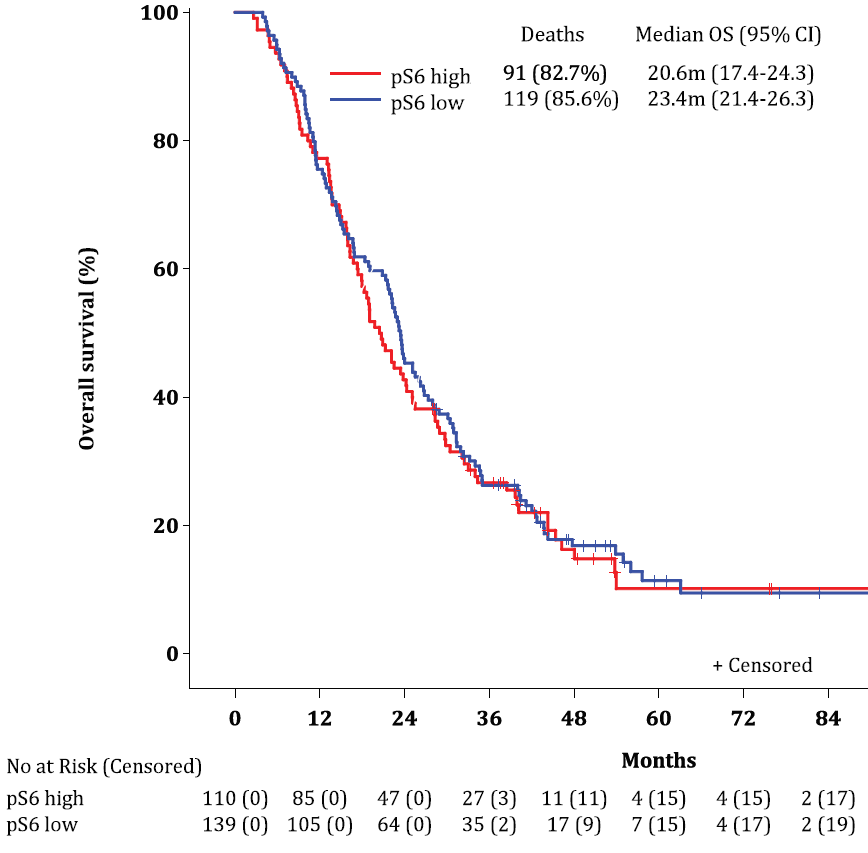


**Figure S4B:** Overall survival (OS), by pS6 status; complete resection

***Notes for Figures S4A-B:***

***Note 1:*** *Interaction p-value (from Cox model including pS6 status with treatment strategy interaction): 0.25.*

***Note 2:*** *Overall median OS was 15.3 months (95%CI: 11.3 – 17.4) for palliative treatment and 22.5 months (95%CI: 19.1 - 24.2) for complete resection.*

***Note3:*** *Log-rank p-value comparing pS6 high vs. low: 0.30 for palliative treatment; 0.70 for complete resection.*

***Note4:*** *There were also 2 patients who haven’t received treatment, not included in this figure.*


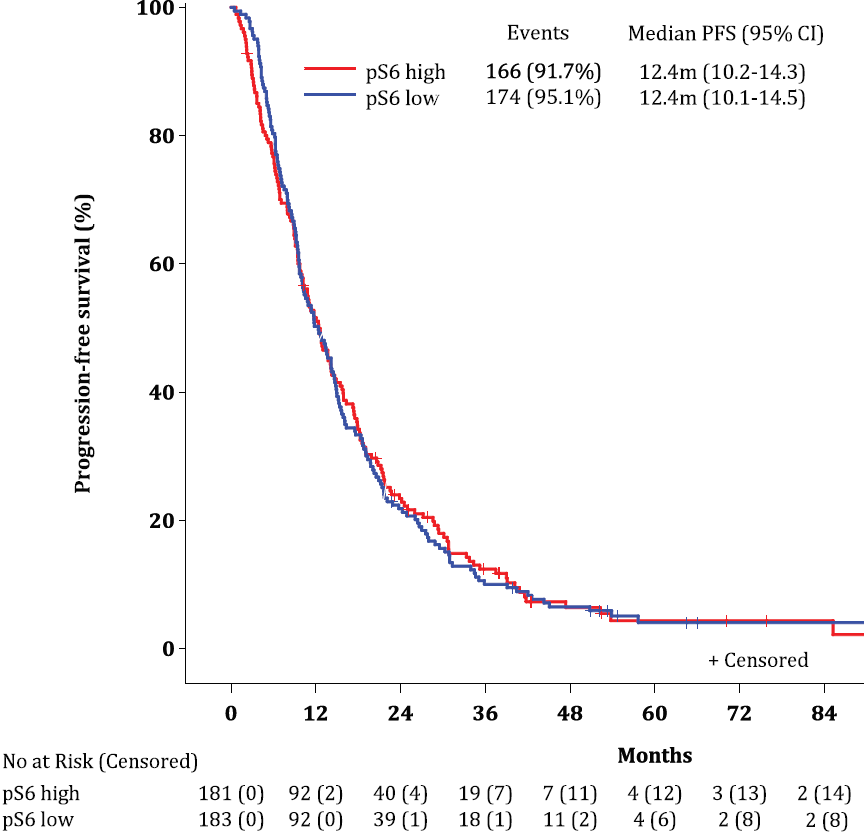


**Figure S5:** Progression-free survival (PFS), by pS6 status

***Note:*** *Log-rank p-value=0.97*


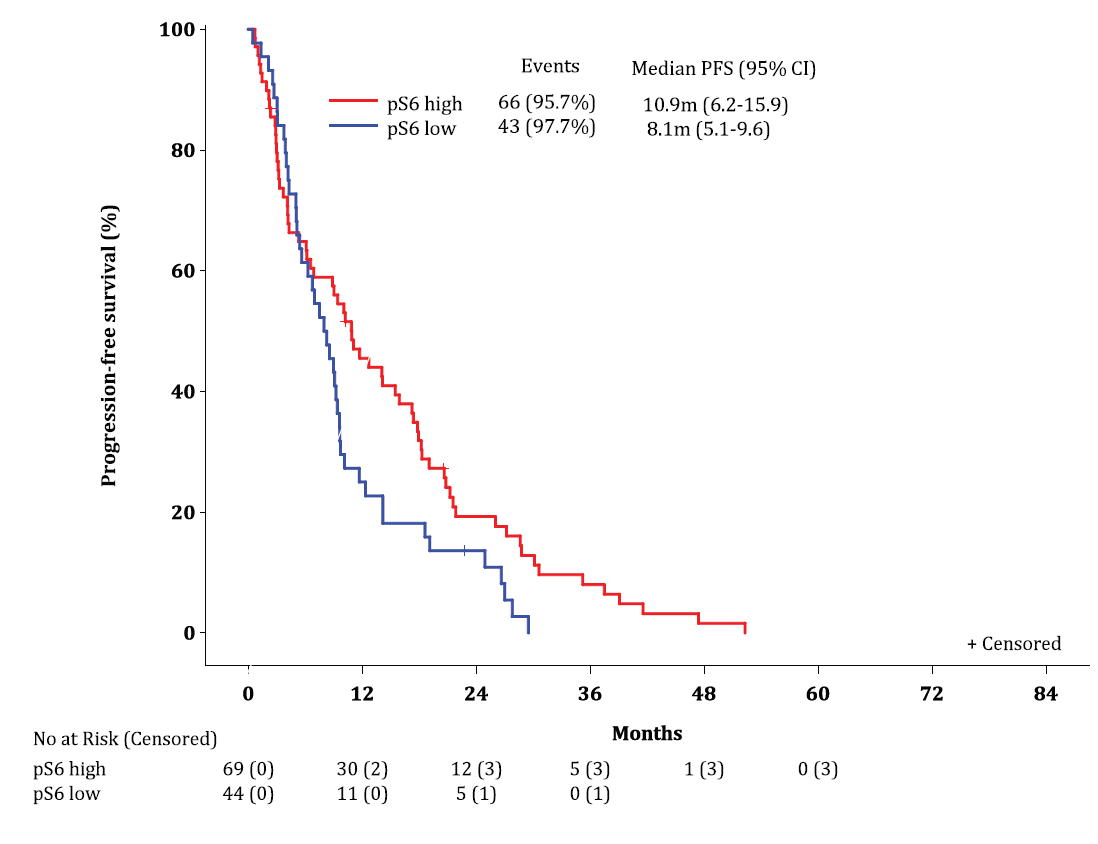


**Figure S6A:** Progression-free survival (PFS), by pS6 status; palliative treatment


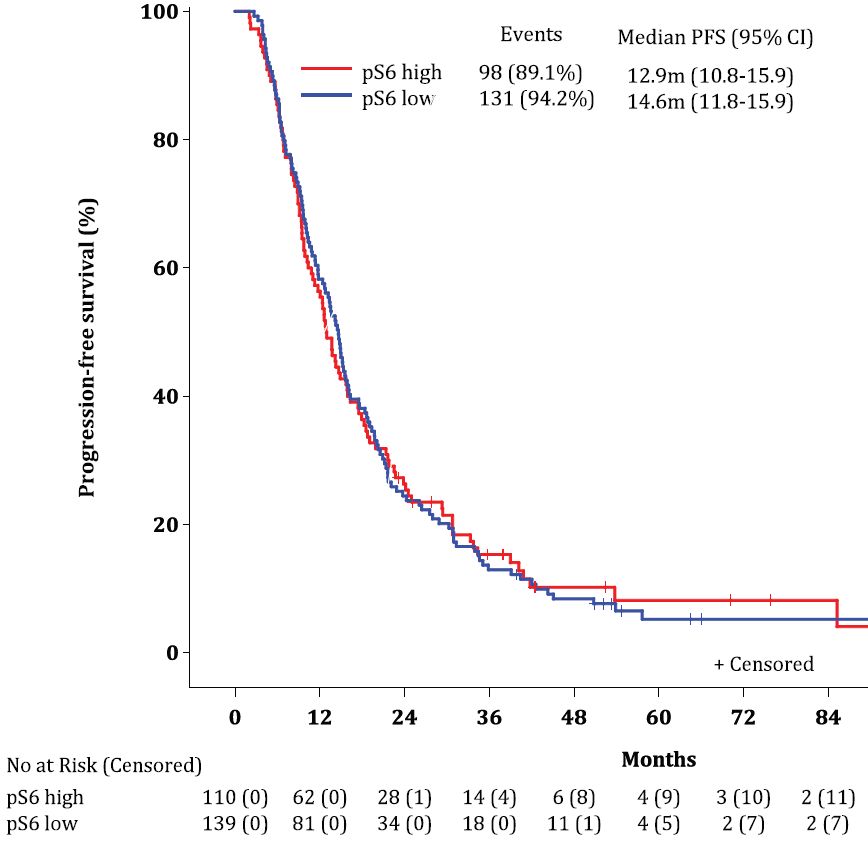


**Figure S6B:** Progression-free survival (PFS), by pS6 status; complete resection

***Notes for Figures S6A-B:***

***Note 1:*** *Interaction p-value (from Cox model including pS6 status with treatment strategy interaction): 0.087.*

***Note 2:*** *Overall median PFS was 9.2 months (95%CI: 6.7 - 10.9) for palliative treatment and 13.8 months (95%CI: 12.4 - 15.3) for complete resection.*

***Note3:*** *Log-rank p-value comparing pS6 high vs. low: 0.045 for palliative treatment; 0.97 for complete resection.*

***Note4:*** *There were also 2 patients who haven’t received treatment, not included in this figure.*


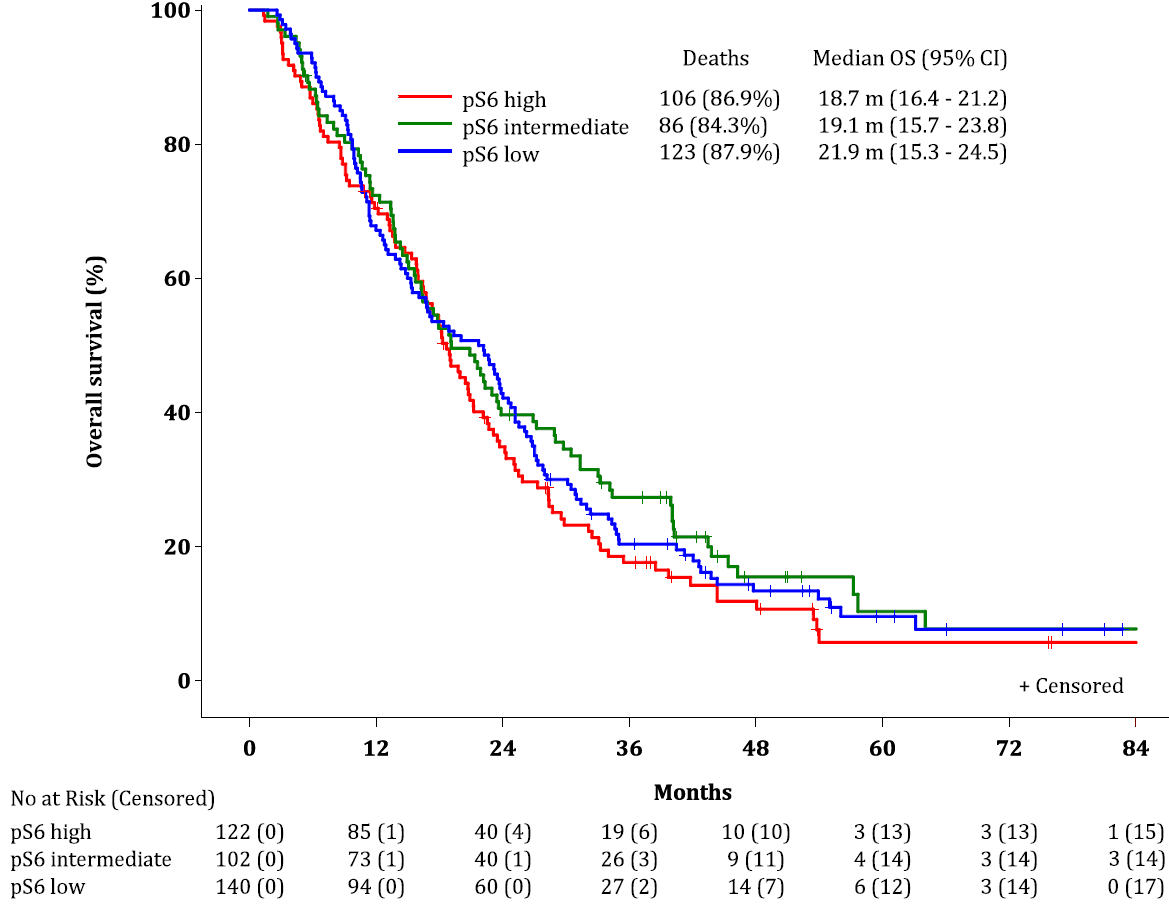


**Figure S7:** Overall survival (OS), by 3-level pS6 status

***Note:*** *Log-rank p-value=0.42*


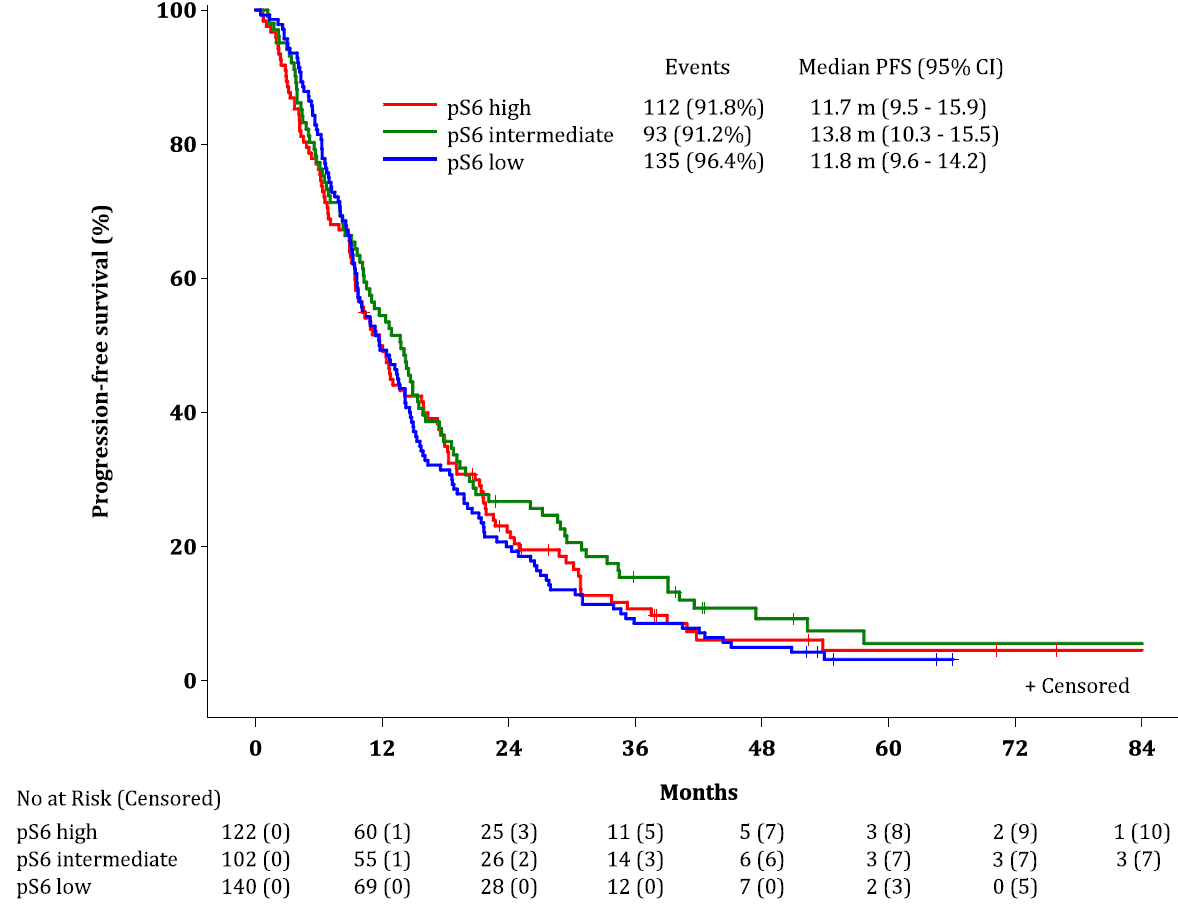


**Figure S8:** Progression-free survival (PFS), by 3-level pS6 status

***Note:*** *Log-rank p-value=0.41*
